# Point Mutations Lead to Increased Levels of c-di-GMP and Phenotypic Changes to the Colony Biofilm Morphology in *Alcanivorax borkumensis* SK2

**DOI:** 10.1264/jsme2.ME18151

**Published:** 2019-02-16

**Authors:** Manoj Prasad, Nozomu Obana, Kaori Sakai, Toshiki Nagakubo, Shun Miyazaki, Masanori Toyofuku, Jacques Fattaccioli, Nobuhiko Nomura, Andrew S. Utada

**Affiliations:** 1 Faculty of Life and Environmental Sciences, University of Tsukuba 1–1–1 Tennodai, Tsukuba, TARA center, Ibaraki 305–8572 Japan; 2 Transborder Medical Research Center, Faculty of Medicine, University of Tsukuba 1–1–1 Tennodai, Tsukuba, Ibaraki 305–8575 Japan; 3 Microbiology Research Center for Sustainability, University of Tsukuba 1–1–1 Tennodai, Tsukuba, Ibaraki 305–8572 Japan; 4 PASTEUR, Département de chimie, École Normale Supérieure, PSL University, Sorbonne Université, CNRS 75005 Paris France; 5 Institut Pierre-Gilles de Gennes pour la Microfluidique 75005 Paris France; 6 School of Life and Environmental Sciences, University of Tsukuba 1–1–1 Tennodai, Tsukuba, TARA center, Ibaraki 305–8572 Japan

**Keywords:** *Alcanivorax*, marine microorganism, hydrocarbonoclastic, c-di-GMP

## Abstract

*Alcanivorax borkumensis* is a ubiquitous marine bacterium that utilizes alkanes as a sole carbon source. We observed two phenotypes in the *A. borkumensis* SK2 type strain: rough (R) and smooth (S) types. The S type exhibited lower motility and higher polysaccharide production than the R type. Full genome sequencing revealed a mutation in the S type involved in cyclic-di-GMP production. The present results suggest that higher c-di-GMP levels in the S type control the biofilm forming behavior of this bacterium in a manner commensurate with other Gram-negative bacteria.

The *Alcanivorax* genus is a ubiquitous group of marine dwelling Gram-negative γ-proteobacteria that metabolize hydrocarbons as their sole carbon source (21, 29). Although present in extremely low numbers in the ocean, during an oil spill, such as the 2010 Deep Water Horizon accident in the Gulf of Mexico, they become one of the most abundant microorganisms in the local environment due to their ecological niche, namely, the ability to exploit readily available hydrocarbons (11, 21). *Alcanivorax borkumensis* is a member of this hydrocarbonoclastic family and is a rod-shaped bacterium that lacks flagella. Since the type strain *A. borkumensis* SK2 was initially isolated and fully sequenced (8, 30), it has been the focus of numerous studies due to its rare biological ability, the novel glycolipid and lipoprotein biosurfactants it produces (2, 18), its technological potential as an agent of bioremediation, and as a model organism to study bacterial adhesion to oil-water interfaces (1).

The ability of *A. borkumensis* to attach at the oil-water interface enables it to form interfacial communities, or biofilms, which also appear to mediate its rapid increase in numbers when consuming spilled oil (21). Most bacteria are known to transition between two states: planktonic, or free-living, and biofilms (17). Biofilms are surface-adhered communities of bacteria covered in self-produced extracellular polymeric substances (EPS) (12, 26, 27), which comprise proteins, DNA, and polysaccharides. EPS encases and protects the community from harsh environmental conditions, while enabling resource sharing.

A deeper understanding of the planktonic-to-biofilm switch in *A. borkumensis* will shed light on its ecological niche and may unlock paths to facilitate its practical utilization as an agent of bioremediation. Clues to this switch may be found by investigating the relationship between cyclic dimeric guanine monophosphate (c-di-GMP) and biofilm formation in other model γ-proteobacteria. The second messenger c-di-GMP is an important regulator of the planktonic-to-biofilm transition in many Gram-negative bacteria. c-di-GMP is synthesized by diguanylate cyclases (DGCs), which have the GGDEF domain, and is degraded by phosphodiesterases (PDEs), which have the EAL or HD-GYP domain (9, 14, 22). In model organisms, such as *Pseudomonas aeruginosa* and *Vibrio cholerae*, the accumulation of intracellular c-di-GMP is accompanied by a decrease in motility and increases in EPS secretion and antibiotic resistance (10). However, unlike these model organisms *A. borkumensis* SK2 lacks flagella and, as a consequence, is unable to actively swim away from a biofilm. Thus, it may require the ability to readily up- or down-regulate biofilm-forming factors, such as EPS synthesis, in order to be able to rapidly adapt to changing resource conditions. *A. borkumensis* forms biofilms at the oil-water interface (5, 21); however, few studies have focused on the relationship between c-di-GMP and biofilm-forming factors in *A. borkumensis* (20). In the present study, we report the discovery of a subpopulation in the type strain *A. borkumensis* SK2 containing three point mutations that alter the colony biofilm morphology and spreading, extracellular polysaccharides, and motility appendage biosynthesis as well as intercellular c-di-GMP levels.

The *A. borkumensis* SK2 bacterial strain was purchased from ATCC (ATCC^®^ 700651^TM^) and cultured in marine broth (Difco Marine Broth 2216) supplemented with pyruvate (10 g L^−1^) as the carbon source following the protocol provided (see Supplementary Fig. S1 for a growth curve). After streaking the bacteria on marine agar (Difco Marine Agar 2216) supplemented with pyruvate (10 g L^−1^) and culturing, the emergence of two distinct colony morphologies was observed: one with rough edges and a greater amount of spreading (R type), and another with smooth edges (S type) and less spreading (Fig. 1A). By counting more than 900 colonies, we note that the ratio of the R to S type was approximately 8:2 (See Supplementary Information [SI] for this method).

The roughness and spreading apparent in R colonies suggested increased motility, while the shiny smoothness of S colonies indicated increased EPS secretion. Differences in the levels of EPS secreted are often observable at the single cell level, with EPS-overproducing cells having a thick layer of EPS covering the cell body (7). To quantify single cell level differences between the R and S types, we initially isolate them using sequential streaking and culturing. We stained for increased levels of cell-associated EPS using India ink, which consists of black colloidal particles suspended in water. Ink added to EPS-abundant cells will stain the culture medium black, but is excluded from a region occupied by the coat of EPS surrounding individual cells (6) (see SI for the method). There were no excluded regions surrounding R-type cells, whereas a thick band surrounds S-type cells (Fig. 1B and C).

To examine the macroscopic effects of this increased level of EPS associated with the cells, we compare qualitative differences in colony biofilm morphologies on motility plates by spotting 2 μL of the stationary phase culture on the center of two separate marine agar plates supplemented with pyruvate. Visible differences rapidly develop between colony morphologies: the edges of the original 2-μL spot of the R type are flatter and rougher in appearance than those of the S type, which develop a smooth and shiny central ring. In the initial few days, we observe the emergence of a central colony (original spot) in both types that is surrounded by two annuli: a thin ring of spreading cells immediately adjacent to the central colony with greater contrast and a larger secondary annulus that was even thinner than the first ring with very low contrast (Fig. 1D and E). Although colony differences in spreading were initially small, the overall morphology significantly changes beyond this point: after approximately 6 d, the R type begins to develop dendritic branches, while the S type remains approximately circular (Fig. 1F and G).

Differences in colony morphologies between the two phenotypes appear to be related to EPS and motility. To test this hypothesis, we transform both phenotypes with fluorescent reporter plasmids (15) containing P*_gap_-gfp*, which constitutively expresses GFP, P*_algA_-gfp*, which expresses GFP when the *algA* promoter is activated, P*_alkB1_*-*gfp*, which expresses GFP in the presence of alkanes, and P*_pilB_*-*gfp*, which expresses GFP when type IV pili are biosynthesized. P*_gap_-gfp* is expressed constitutively and emission, as measured using the Varioskan Flash plate reader (ThermoFisher, Waltham, MA, USA), indicates no significant differences in the expression of GFP between R and S. Regarding *algA*, although its function is hypothetical in *A. borkumensis*, *algA* in *P. aeruginosa* is known to be involved in alginate biosynthesis (3, 4, 13). Following an overnight culture in ONR7a minimal medium supplemented with pyruvate (10 g L^−1^), we observe markedly higher levels of fluorescence in P*_algA_-gfp* S-type cells than in the R type; this indicates increased *algA* activity and imples upregulation of alginate synthesis in the S type. We also find significantly higher levels of fluorescence in P*_pilB_-gfp* R-type cells relative to the S type, which corroborates our results of higher motility in the R type (Fig. 2A). In *P. aeruginosa*, the ATPase PilB is known to assemble the pilus fiber, which enables ‘twitching’ surface motility (28).

To characterize differences between the two strains in different carbon sources, we cultured the reporter strains in ONR7a supplemented with hexadecane, instead of pyruvate. We use hexadecane as a model carbon source to represent the *n*-alkanes found in spilled oil (16, 29). We add 100 μL of oil to 4 mL of medium (85 mM hexadecane), which is similar to the molar concentration of pyruvate used in these experiments. When cultured on hexadecane, the R type exhibit marked increases in *algA* activity and a simultaneous decrease in *pilB* activity. Under the same conditions, we observe similar, but markedly smaller changes in the S type. In contrast, no marked changes are found in *alkB1* activity in either strain. These results imply that the R type modulates its responses to different growth substrates, while the S type appears to lack this ability.

To clarify the origin of these phenotypic differences, we sequenced the entire genome of both types using next generation sequencing. We generated an average of 23,533,138 mapped reads at a mean depth of 734 per sample, finding a number of shared point mutations in the R and S types relative to the reference sequence (8, 21) (see SI for the method and Supplementary Table S1). We identify three additional point mutations between the R and S types. Interestingly, a mutation was detected in the gene ABO_2691 in the S type only, which encodes for a protein with GGDEF and EAL domains (Table 1). This mutation suggest that c-di-GMP regulation may be involved in the phenotypic changes observed in the S type (see Supplementary Fig. S2 for a schematic of the locations of the point mutations). Higher intracellular levels of c-di-GMP correlate with increased levels of EPS and decreased levels of motility in a wide range of bacteria, including *Escherichia coli*, *P. aeruginosa*, *Salmonella enterica* serovar Typhimurium, and *V. cholerae* (9, 23–25). To directly confirm the effects of the mutation in ABO_2691, we quantify intracellular c-di-GMP levels from the R and S types using LC-MS (Shimadzu, Tokyo, Japan) (see SI and Supplementary Fig. S3) (19). The results obtained reveal that when grown on pyruvate, intracellular concentrations of c-di-GMP in the S type are more than 2-fold higher than those in the R type. We also note that c-di-GMP concentrations in the R type are higher when grown on hexadecane than on pyruvate (see Fig. 2B). In contrast to the increase observed in the R type, the responses of the S type under both conditions are weaker. We find that the biofilm cells of the model organism *P. aeruginosa* PAO1 contain an approximately 2-fold higher amount of c-di-GMP than that found in planktonic cells (25). This is similar to the difference we observe between the S and R types, whereas the absolute quantities of c-di-GMP markedly differ.

In the present study, we describe two phenotypes in the *A. borkumensis* SK2 stock strain that arise due to the presence of three point mutations, which appear to increase the levels of intercellular c-di-GMP. These results show that the R type approximately phenocopies the S type when cultured on hexadecane, indicating a relationship between the assimilation of aliphatic compounds, c-di-GMP accumulation, and the up-regulation of factors leading to biofilm formation. Since aliphatic compounds are immiscible with water, the switch from planktonic to biofilm at the oil-water interface may be required for this bacterium to remain localized around its growth substrate. This is in contrast to other bacteria whose growth substrate is dissolved in the aqueous environment. Due to this difference and in consideration of the ratio of appearance, we consider the R type to represent the WT, which may modulate its behavior in response to the available carbon source, while the S type appears to be deficient in this ability. In the environment, these two populations may provide an ecological flexibility to exploiting new resources. Depending on the type and availability of the carbon source, one population may more readily exploit a resource by quickly forming a biofilm, while the other may more judiciously tune its response this dual nature may enable rapid proliferation and effective dispersal from a spent source.

## Supplementary Information



## Figures and Tables

**Fig. 1:**
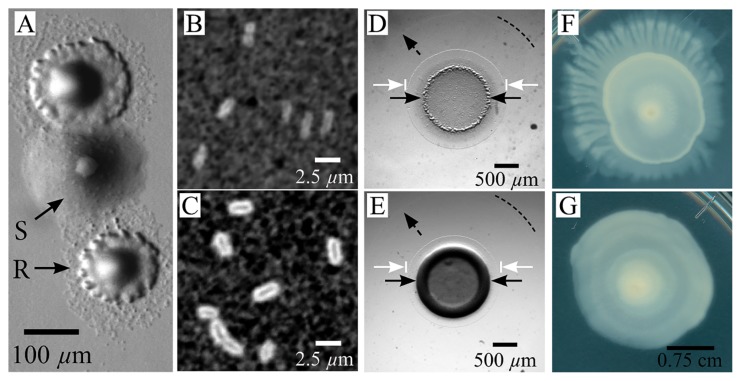
Phenotypic differences between R and S types. (A) Marine agar plate with two different phenotypes growing from the same stock. Colonies were grown on marine agar supplemented with 10 g L^−1^ pyruvate. (B) India ink exclusion staining of the secreted extracellular polysaccharide in the R type and (C) S type cells taken from a stationary phase liquid culture mixed in a 1:1 ratio with the ink. (D and E) Morphological differences between (D) R type and (E) S type cells after 2 μL of 0.01 OD_600nm_ liquid culture was deposited and cultured at 30°C for 24 h. In (D and E), the solid black arrows indicate the central colony, the white arrows show the edges of the first annular band of cells surrounding the central colony, and the dashed arrow and line are guides indicating the outer edge of the second annular band of spreading cells. (F) A 5-d-old R colony displaying a dendritic structure. (G) A 5-d-old S colony showing a circular symmetry. The scale bar in (G) corresponds to (F) and (G).

**Fig. 2:**
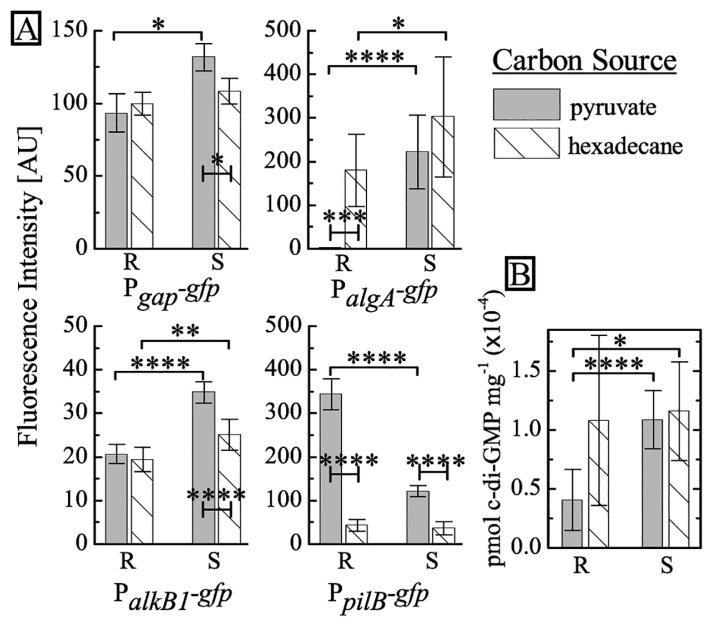
(A) Fluorescence intensities for stationary phase cultures grown in ONR7a supplemented with different carbon sources in R and S types harboring P*_gap_-gfp*, P*_algA_-gfp*, P*_alkB1_-gfp*, and P*_pilB_-gfp* plasmids. Intensities are normalized by the respective OD_600nm_ values from each culture and background fluorescence is subtracted using auto-fluorescence from the strains harboring the empty vector (pPROBE-GT). Cultures were grown on pyruvate (gray) and hexadecane (lines). Error bars represent the standard deviation of measurements performed in triplicate. (B) Intracellular c-di-GMP concentrations measured by LC-MS. c-di-GMP was extracted from whole cells and the quantification method is described in SI. Paired *t*-tests were performed for statistical analyses. * denotes *P*<0.05, ** denotes *P*<0.01, *** denotes *P*<0.001, and **** denotes *P*<0.0001. R and S types grown in their respective carbon sources show no significant difference. Error bars represent the standard deviation with *n*=8 for R in pyruvate, *n*=6 for R in hexadecane, *n*=8 for S in pyruvate, and *n*=5 for S in hexadecane.

**Table 1:** Point mutations discovered between R and S types after full genome sequencing.

Position	Locus tag	Gene Name	Strand	Pos. in gene	Ref.	Rough	Smooth	Product	Mutation
1253293	ABO_1918	*acdS* (ABO_1918)	+	548	G	G	Ins AGGTG	butyryl-CoA dehydrogenase	Shorten ORF
2460898	ABO_2154	*degU* (ABO_2154)	+	326	T	A	T	transcriptional regulatory protein degU	V109E
3041608	ABO_2691		+	2795	G	G	A	sensory box protein/GGDEF, EAL domain protein	G932D
