# Supplementary material for: Point Mutations Lead to Increased Levels of c-di-GMP and Phenotypic Changes to the Colony Biofilm Morphology in Alcanivorax borkumensis SK2
Source: Microbes Environ. 2019 Feb 16;34(1):104–7. doi: 10.1264/jsme2.ME18151 (PMC6440734; doi:10.1264/jsme2.ME18151)

Title: Point mutations lead to increased levels of c-di-GMP and phenotypic changes to colony biofilm morphology in *Alcanivorax borkumensis* SK2

Author's names

Manoj Prasad[1†], Nozomu Obana[2,3†], Kaori Sakai[4,5], Toshiki Nagakubo[1], Shun Miyazaki[6], Masanori Toyofuku[1,3], Jacques Fattaccioli[4,5], Nobuhiko Nomura[1,3]*, Andrew S. Utada[1,3]*

Supplementary Information

Percentages of R and S type: To estimate the ratio of appearance of R type to S types, we streaked 8 plates on marine agar supplemented with pyruvate from the original stock and cultured for 48 h. We took 5 representative images from each plate and counted the number of each type of colony. We counted a total of 933 colonies, where 778 were R colonies and 155 were S colonies. The average and standard deviation of the percentage on these 8 plates for R and S types are 83 ± 6% and 17 ± 6%, respectively.

India Ink Exclusion Staining: To image the excluded region surrounding the S type cells, we mix 2 μL of bacterial culture with 2 μL of commercially available Bokueki Sumi Ink (Sun Note, Osaka, Japan) on a glass slide. We then cover the sample with cover glass and image using phase contrast microscopy at 100x magnification.

Full genome resequencing: We extracted genomic DNAs from S- and R-type cells using QIAGEN Genomic-tip 100/G (Qiagen, Valencia, CA, USA) according to manufacturer's instructions. Next generation sequencing and data analysis were supported by Macrogen Japan Co. (Macrogen Japan, Tokyo, Japan). The libraries were prepared using TruSeq DNA

PCR-free kit (Illumina, San Diego, CA, USA). Each sample was sequenced using 100-bp paired end reads using the Illumina platform. The reads were mapped to 100% of the reference sequence *A. borkumensis* SK2 genome (Genbank accession number AM286690.1) at a mean depth of over 620. We found 9 SNPs at a depth of at least 433 using SAMTools.

<u>Quantification of c-di-GMP by LC-MS:</u> Cells were grown in ONR7Aa for two days and then sub-cultured for another two days to reach stationary phase. Bacteria were collected from 8 mL of culture media, washed, then lysed using sonication (Biorupter, Tokyo, Japan). c-di-GMP was extracted using a previously reported protocol (26). Briefly, we washed the lysate in 63% v/v ethanol in phosphate buffered saline (PBS) twice and kept the supernatant. We then evaporated the supernatant containing the c-di-GMP overnight under vacuum. The residue was stored at -80° C until ready to be measured. The residue was resuspended in 100 μL of DI water. We quantified the amount of c-di-GMP with an LC-ESI-MS with a Shimadzu Nexera X2 system equipped with a XSelect HSS C18 column, 4.6 x 150 mm (Waters, Milford MA, USA) under the following conditions: column temperature, 40° C; gradient elution; mobile-phase solvent A [10mM ammonium acetate in DI H_2O] and solvent B [methanol]; mobile-phase composition, 0% solvent B at 0-10 min, 0-30% solvent B at

18-20 min, 30-0% solvent B at 20-24 min, and 0% solvent B at 24-30 min; and flow rate, 0.5 mL min^{-1}; and photodiode array detector, 190~600 nm. We obtained the MS analysis data using a mass spectrometer with ESI in the positive ion multiple reaction monitoring mode. The monitored product ions were *m/z* 248, *m/z* 540, and *m/z* 152. We used commercially available cyclic-di-GMP (CAS No. 61093-23-0) with a purity of ≥ 95%, purchased from Cayman Chemical (Ann Arbor, MI, USA) as a reference for the LC-MS. We quantify the amount of protein per sample after sonication with the Pierce bicinchoninic acid (BCA) protein assay kit (ThermoFisher, Waltham MA, USA) and measure the output using Varioskan Flash plate reader. The amount of c-di-GMP was normalized by the amount of protein.

Supplementary Table S1. Point mutations between the *A. borkumensis* SK2 used in this study compared to the reference sequence.

Position	Locus Tag	Gene Name	Strand in gene	Pos. change	Nucleotide	Product	Mutation
96133	ABO_0091		+	20	C>G	Conserved hypothetical membrane protein	A7G
114570	ABO_0106		−	138	T>C	Twitching motility protein PilJ	M631V
634016	ABO_0575		−	84	Del A	oxidoreductase, aldo/keto reductase family	Shorten ORF
2291109	ABO_2004		−	13	Ins C	conserved hypothetical protein	Generate ABO_2004-2003 fusion
2524864	ABO_2211		+	1021	A>G	thiamine phosphate phosphatase	N341D
2588003	ABO_2267		−	458	C>A	conserved hypothetical protein	G360V

Supplementary Figure Legends

Supplementary Fig S1. Growth curves of R type (♦) and S type (○) in marine broth supplemented with 10 g L^{-1} pyruvate. Error bars represent the standard deviation of the optical density in triplicate at OD_{600nm}.

Supplemental Figure: 1
Author: M. Prasad

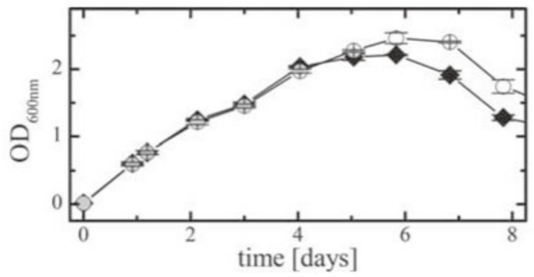

Supplementary Fig S2. Diagram of the location of the point mutations. The vertical arrow indicates the location within the labeled gene.

Supplemental Figure: 2
Author: M. Prasad

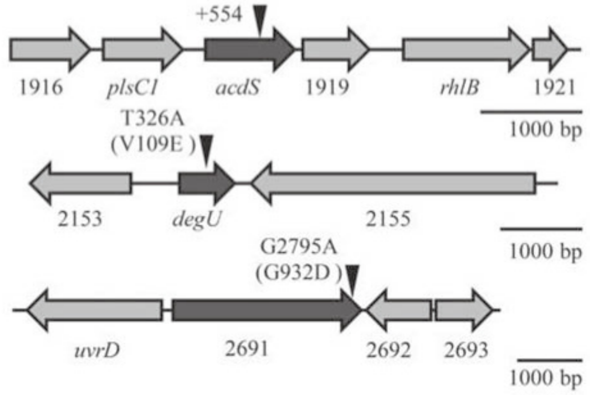

Supplementary Fig S3. Cyclic-di-GMP LC-MS chromatograms. C-di-GMP was extracted from R and S type cells grown in either pyruvate or hexadecane, then analyzed by LC-MS. Two product ions with *m/z* 152.15 (black lines) and *m/z* 540.05 (gray lines) derived from a precursor ion of c-di-GMP with *m/z* 690.90 were detected in the positive ion mode.

Supplemental Figure: 3
Author: M. Prasad

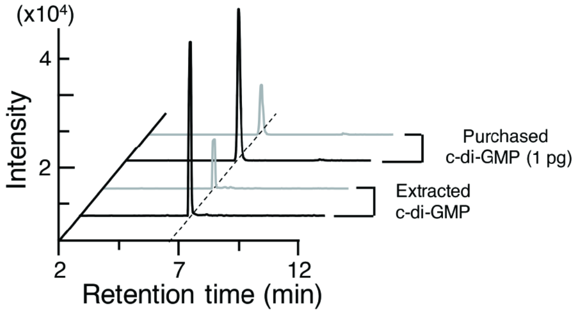

Supplement: Supplementary file 1 [file 34_104_s1.pdf]
